# Environmental exposure to per- and polyfluoroalkyl substances and childhood congenital heart disease: a mixed analysis

**DOI:** 10.3389/fpubh.2025.1657168

**Published:** 2025-11-27

**Authors:** Xianting Jiao, Liqing Zhao, Yuejuan Xu, Jiawei Gao, Weifeng Tang, Yurong Wu, Ling Yang, Jihong Huang, Yi Guo, Kun Sun, Sun Chen

**Affiliations:** 1 Department of Pediatric Infectious, Department of Pediatric Cardiology, Xinhua Hospital Affiliated to Shanghai Jiao Tong University School of Medicine, Shanghai, China; 2 Department of Pediatric Cardiology, Xinhua Hospital Affiliated to Shanghai Jiao Tong University School of Medicine, Shanghai, China; 3 Ministry of Education-Shanghai Key Laboratory of Children’s Environmental Health, Xinhua Hospital, Shanghai Jiao Tong University School of Medicine, Shanghai, China; 4 Center for Reproductive Medicine, Shanghai First Maternity and Infant Hospital, School of Medicine, Tongji University, Shanghai, China

**Keywords:** per- and polyfluoroalkyl substances, congenital heart disease, mixed analysis, Bayesian kernel-machine regression, weighted quantile sum

## Abstract

**Background:**

Per- and polyfluoroalkyl substances (PFAS) exposure is associated with various health risks. However, limited research has explored their potential connection with congenital heart disease (CHD).

**Objective:**

This study aimed to investigate the relationship between different types of PFAS and childhood CHD using a mixed analysis approach.

**Methods:**

A hospital-based case–control study was conducted involving 282 children with CHD and 282 control participants. Plasma samples were analyzed for 19 PFAS congeners. Logistic regression, Bayesian kernel-machine regression (BKMR), and weighted quantile sum (WQS) models were employed to assess the association between individual PFAS and PFAS mixtures with CHD risk.

**Results:**

Analysis of 564 subjects revealed higher plasma concentrations of various PFAS compounds in the CHD-group. Logistic regression identified significant associations between CHD risk and specific PFAS, notably 6:2 Chlorinated polyfluoroalkyl ether sulfonic acid (6:2 Cl-PFESA) (OR = 1.65; 95%CI: 1.40–1.94), 8:2 8:2 Cl-PFESA (OR = 1.69; 95%CI: 1.69–2.19), Perfluorobutanoic acid (PFBA) (OR = 2.68; 95%CI: 2.28–3.15), Perfluorobutane sulfonic acid (PFBS) (OR = 2.38; 95%CI: 1.77–3.22), Perfluorohexanoic acid (PFHxA) (OR = 2.62; 95%CI: 1.99–3.45), and Perfluorotetradecanoic acid (PFTeDA) (OR = 2.18; 95%CI: 1.81–2.63). BKMR analysis confirmed these findings. WQS analysis emphasized PFBA, 8:2 Cl-PFESA, PFHxA, and PFTeDA as key contributors to the association between PFAS mixture exposure and CHD risk.

**Conclusion:**

Exposure to PFAS mixtures was associated with an increased risk of CHD in children, with 6:2 Cl-PFESA, 8:2 ClPFESA, PFBA, PFHxA, and PFTeDA playing significant roles.

## 1 Introduction

Per- and polyfluoroalkyl substances (PFAS) stand out among synthetic chemicals for their unique properties, including resistance to water and grease (1, 2). These characteristics have made them essential in a wide range of industrial and consumer products (1, 3). However, the pervasive use of PFAS has led to their widespread presence in the environment, sparking concerns over their persistence, tendency for bioaccumulation, and the potential for adverse health effects (4–7). Emerging research underscores associations between PFAS exposure and various health risks, encompassing cancer, immune dysfunction, reproductive disorders, liver damage, endocrine disruption, and developmental effects (4, 7–10). Additionally, evidence highlights PFAS’s link to cardiovascular disease (CVD) in adults (11, 12), yet further investigation is needed to discern its impact, particularly on congenital heart disease (CHD) in children.

CHD encompasses a spectrum of structural heart anomalies present from birth, affecting approximately 1% of newborns worldwide (13, 14). The ramifications of CHD are significant, impacting not only patients but also their families and healthcare systems (15). The condition often necessitates surgical and long-term medical interventions and is associated with numerous comorbidities. Identifying environmental contributors to CHD is imperative for crafting effective prevention and public health measures. PFAS, as environmental endocrine disruptors, may interfere with normal endocrine function, particularly during crucial stages of fetal heart development, potentially resulting in structural and congenital heart anomalies (12, 16, 17). Mechanisms may involve alterations in hormonal pathways, disruption of cellular signaling, and changes in gene expression crucial for heart development (17–19).

While existing research has provided valuable insights into the association between PFAS exposure and CVD in adults, there remains a substantial gap in understanding the impact of PFAS on childhood CHD (12, 20, 21). Prior studies have predominantly focused on legacy PFAS compounds, employing simplistic regression analysis methods that fail to comprehensively explore the complex mixture of PFAS exposure (22–24). Moreover, the existing research has largely overlooked the potential distinct or heightened effects of newer alternatives and branched isomers of PFAS. This oversight is concerning given the evolving landscape of PFAS usage, where traditional compounds are being phased out in favor of alternatives with yet-to-be-fully-understood long-term health impacts (25, 26).

Recognizing these limitations, there is an urgent need for more comprehensive investigations into the broad spectrum of PFAS compounds and their roles in influencing childhood CHD. Therefore, this study aims to address this gap through a hospital-based case–control design, employing innovative statistical methods to explore mixed exposure to PFAS. By elucidating the main contributors to these associations, our study seeks to uncover the environmental determinants of childhood CHD, providing insights crucial for informing future preventive strategies and public health interventions.

## 2 Methods and materials

### 2.1 Study population

Our research focused on children diagnosed with CHD and matched controls without CHD, recruited from Xinhua Hospital affiliated to Shanghai Jiao Tong University School of Medicine in China. The CHD group was recruited from cases that underwent cardiac surgery in our pediatric heart center between June 2021 and September 2023. The recruitment process and blood collection were done before the cardiac surgery to avoid the perioperative impact on samples, such as blood transfusion. The inclusion criteria for the CHD group were all types of congenital heart defects, excluding cases associated with other malformations or known chromosomal abnormalities.

The control group was recruited from the outpatient for children’s healthcare during the same period. The inclusion criteria for the control group were healthy children without known systemic diseases or chromosomal abnormalities. The cardiac evaluation was determined by transthoracic echocardiography in a tertiary hospital. Nearly twice the number of the control group was recruited to ensure a sufficient number for matching the case group. After recruitment, an equal number of participants were matched to balance the case and control groups based on key demographic factors (age, sex and race). Supplementary Figure S1 outlines the recruitment process.

A total of 282 children with CHD and an equal number of controls were selected through rigorous screening and clinical evaluations, ensuring demographic (age and sex) compatibility to strengthen the study’s validity. Informed consent was obtained from the parents or guardians of all participants. The study protocol was approved by the ethics committee of Xinhua Hospital (Approval no. XHEC-NSFC-2022-070).

### 2.2 PFAS measurements

Upon enrollment, blood samples were collected and immediately processed, then stored at −80 °C to maintain integrity and prevent degradation. We analyzed these samples for a panel of 19 PFAS congeners, including traditional and emerging PFAS, using ultra-performance liquid chromatography (UPLC) paired with triple quadrupole tandem mass spectrometry (MS/MS). This method provided the sensitivity and specificity required for precise PFAS quantification. Reference standards for all PFAS were sourced from Wellington Laboratories to ensure measurement accuracy (see Table 1).

**Table 1:** Overview of the 19 selected PFAS.

PFAS	Chemical full name	Internal Standard	LOD (ng/mL)	LOQ (ng/mL)
6:2 ClPFESA	6:2 Chlorinated polyfluoroalkyl ether sulfonic acid	M8PFOS	0.0008	0.0025
8:2 ClPFESA	8:2 Chlorinated polyfluoroalkyl ether sulfonic acid	M8PFOS	0.0004	0.0012
FOSA	Perfluorooctane sulfonic acid	M8FOSA	0.0010	0.0032
PFBA	Perfluorobutanoic acid	MPFBA	0.0025	0.0080
PFBS	Perfluorobutane sulfonic acid	MPFBS	0.0050	0.0159
PFDA	Perfluorodecanoic acid	M6PFDA	0.0061	0.0194
PFHpS	Perfluoroheptane sulfonic acid	M3PFHxS	0.0007	0.0022
PFHxA	Perfluorohexanoic acid	M5PFHxA	0.0017	0.0054
n-PFHxS	Perfluorohexane sulfonic acid	M3PFHxS	0.0035	0.0111
PFNA	Perfluorononanoic acid	M9PFNA	0.0052	0.0166
PFOA	Perfluorooctanoic acid	M8PFOA	0.0045	0.0145
n-PFOS	Perfluorooctane sulfonic acid	M8PFOS	0.0035	0.0111
PFPeA	Perfluoropentanoic acid	M5PFPeA	0.0017	0.0054
PFTeDA	Perfluorotetradecanoic acid	M2FTeDA	0.0090	0.0287
PFUdA	Perfluoroundecanoic acid	M7PFUDA	0.0100	0.0318
6 m-PFOS	6-mono-perfluorooctane sulfonic acid	M8PFOS	0.0050	0.0159
3,4,5 m-PFOS	3,4,5-mono-perfluorooctane sulfonic acid	M8PFOS	0.0086	0.0274
1 m-PFOS	1-mono-perfluorooctane sulfonic acid	M8PFOS	0.0012	0.0038
Br-PFHxS	Brominated perfluorohexane sulfonic acid	M3PFHxS	0.0095	0.0301

PFAS, Per- and polyfluoroalkyl substances; LOD, Limit of Detection; LOQ, Limit of Quantification.

### 2.3 Pretreatment of PFAS

Before assessing PFAS levels, we implemented a detailed pretreatment protocol. Briefly, a 0.1 mL plasma was spiked with 2 ng internal standard mixture, then 1 mL of 0.1 M formic acid was used. Sample extraction and cleanup were performed using Oasis-HLB cartridges (3 mL/150 mg; Waters Inc., Milford, MA, USA). Before sample loading, the solid-phase extraction (SPE) cartridges were conditioned with 1 mL MeOH followed by 1 mL of 0.1 M formic acid. The plasma samples were then loaded onto cartridges, rinsed with 1 mL of 0.1 M formic, 3 mL 0.1 M formic acid/MeOH (4:1, v/v) and 0.5 mL water (1% ammonium acetate). After 30 min of vacuum dried, the target substances were eluted with 1.8 mL of ACN (1% ammonium acetate). The final eluent was collected, concentrated and then reconstituted with 0.1 mL MeOH/water (7:3; v/v) for ultra-performance liquid chromatography–tandem mass spectrometry (UPLC-MS/MS) analysis. This procedure, validated by calibration curves showing excellent linearity (*R*^2^ > 0.99), ensured the removal of impurities, enhancing subsequent PFAS measurement accuracy and reliability. The obtained matrix spiked recoveries ranged from 81.5% (PFDS) to 116% (N-EtFOSAA). The limits of detection (LODs) ranged from 0.0005 for Σ3,4,5 m-PFOS to 0.012 ng/mL for PFHpA.

### 2.4 Instrument analysis

Instrumental analysis employed UPLC-MS/MS, specifically the Agilent 1,290 Infinity Series HPLC system paired with the Agilent 6,495 C triple quadrupole mass spectrometer. Operated in electrospray ionization (ESI) negative ion mode, this setup optimized sensitivity and precision. Chromatographic separation was achieved using a ZORBAX RRHD Eclipse Plus C18 column, complemented by a ZORBAX Eclipse Plus C18 guard column, with conditions maintained at 40 °C. A precise injection volume of 0.005 mL and a gradient elution program involving milli-Q water with ammonium acetate and methanol facilitated efficient separation of PFAS congeners. The source temperature was set at 375 °C and flow of the sheath gas15 L min^−1^. The nebulizer gas was set at 35 psi and the capillary voltage was −3,500 V. The mass spectrometer was performed in the electrospray ionization (ESI) negative ion multiple reaction monitoring (MRM) mode.

### 2.5 Quality assurance and quality control

Our study maintained rigorous quality assurance and control (QA/QC) protocols to ensure analytical accuracy. We regularly introduced solvent blanks and analyzed control samples to monitor instrument impurity and background contamination. For every 21 samples, one procedural blank (ultrapure water), one matrix blank (sheep plasma) and two matrix spiked controls (1 ng/mL and 10 ng/mL) were checked. The final concentration of PFAS was determined by subtracting the level of matrix blanks. Quality control samples at low and high spiked levels were periodically analyzed to assess method stability. Comprehensive precautions, including the use of methanol-rinsed polypropylene tubes and continuous solvent injection, minimized potential contamination. We established strict inclusion criteria for PFAS congeners based on detection rates and set precise limits of detection (LOD) and limit of quantification (LOQ), ensuring reliable concentration estimates for PFAS analysis. The LODs for PFAS ranged from 0.0003 to 0.015 ng/mL. Recoveries for all target compounds were within 82.0–118.2%, and relative standard deviations (RSDs) were < 20%. These exhaustive QA/QC measures reinforced the study’s analytical integrity, contributing to the generation of high-quality data for informed scientific conclusions.

### 2.6 Statistical analysis

In our study, we employed standard statistical methodologies to explore the intricate relationships between PFAS exposure and the risk of CHD in children. Initially, we conducted descriptive statistical analysis, presenting continuous variables as medians with interquartile ranges and categorical variables as frequencies and percentages. Differences in PFAS levels across groups were evaluated using the Wilcoxon rank-sum test, while categorical variables underwent analysis using the chi-square test. To ensure data comparability and account for potential non-linear relationships, PFAS concentration data were transformed using a logarithmic scale. Spearman’s correlation coefficients were then utilized to assess correlations among log-transformed PFAS concentrations.

To investigate the association between individual PFAS exposures and the risk of CHD, logistic regression analysis was employed, adjusting for age and sex. Given the potential for non-linear associations, we utilized restricted cubic spline (RCS) analysis with three strategically placed nodes. This approach allows for flexible modeling of non-linear relationships by fitting cubic polynomial functions within defined intervals of the predictor variable range (27). By incorporating logistic regression and RCS analysis, we conducted a comprehensive examination of the complex dynamics between PFAS exposure and CHD risk, adjusting for relevant covariates to ensure statistical robustness.

We applied the Bayesian Kernel Machine Regression (BKMR) model to assess the health impacts of mixed PFAS exposures. BKMR is adept at elucidating individual and combined effects of multiple pollutants on health outcomes, capturing potentially non-linear and interactive exposure relationships using a kernel function (28). The BKMR model’s reliability was ensured through the use of 20,000 MCMC iterations, which serve as an internal cross-validation mechanism, assessing the stability and consistency of our findings. PFAS data were log-transformed and standardized (z-score normalization) to ensure comparability. The BKMR model, adjusted for covariates such as age and sex, utilized the Posterior Inclusion Probability (PIP) criterion to determine the significance of specific PFAS compounds in relation to CHD risk, with parameter estimation achieved through 20,000 Markov Chain Monte Carlo (MCMC) iterations.

Furthermore, we explored the Weighted Quantile Sum (WQS) regression method to examine the collective impact of mixed exposures. By categorizing PFAS concentrations into quartiles and assigning weights to each exposure within a WQS index, this method quantified each compound’s contribution to CHD risk (29). The reliability of estimated weights was validated through 200 bootstrap samples, each subjected to regression analysis to discern the WQS index’s association with CHD risk. Adjustments for covariates such as age and sex were made to provide insights into the cumulative effect of PFAS exposure on CHD risk.

All statistical analyses were conducted using R software, with the “rms,” “bkmr,” and “gWQS” packages utilized for model implementations. Statistical significance was established at a *p*-value of less than 0.05 (two-tailed), ensuring the scientific rigor and reliability of our findings.

## 3 Results

### 3.1 Descriptive analysis

A total of 564 subjects were included in this study, consisting of 282 individuals in the CHD group and 282 in the control group (Supplementary Table S1). The mean age of the population was 7.3 years (±2.3), with a slightly lower mean age observed in the CHD group (7.1 ± 2.5 years) compared to the control group (7.5 ± 2.1 years). Gender distribution was comparable between both groups, with 62.1% boys and 37.9% girls overall, and nearly equal proportions within each group: 63.1% boys and 36.9% girls in the CHD group, compared to 61.0% boys and 39.0% girls in the control group. This demographic balance minimizes potential confounding variables related to age and sex, providing a robust foundation for subsequent exposure risk assessment. Some PFAS concentrations exhibited significant correlations with each other, with Spearman correlation coefficients ranging from −0.09 to 0.93 (Supplementary Figure S2).

Table 2 presents a comprehensive comparison of plasma PFAS levels between CHD cases and controls. Significant differences between the two groups are apparent for several PFAS compounds. For instance, 6:2 Cl-PFESA levels are notably higher in the CHD group (*p* < 0.001), with a mean of 2.789 ng/mL compared to 1.091 ng/mL in controls. Similarly, PFBA shows a pronounced disparity, with a CHD group mean of 0.223 ng/mL against a mean of 0.024 ng/mL in the control group (*p* < 0.001). This trend of higher PFAS concentrations in the CHD group is also evident with 8:2 ClPFESA (*p* < 0.001), PFBS (*p* < 0.001), PFHxA (*p* < 0.001), PFPeA (Perfluoropentanoic acid) (*p* < 0.001), and PFTeDA (*p* < 0.001), among others, all exhibiting statistically significant differences. Conversely, certain compounds such as PFDA (Perfluorodecanoic acid)(*p* = 0.197), n-PFHxS (Perfluorohexane sulfonic acid) (*p* = 0.810), PFNA (Perfluorononanoic acid) (*p* = 0.220), n-PFOS (Perfluorooctane sulfonic acid) (*p* = 0.095), PFUdA (Perfluoroundecanoic acid) (*p* = 0.761), 6 m-PFOS (6-mono-perfluorooctane sulfonic acid) (*p* = 0.187), 345 m-PFOS (3,4,5-mono-perfluorooctane sulfonic acid) (*p* = 0.958), and 1 m-PFOS (1-mono-perfluorooctane sulfonic acid) (*p* = 0.030) did not show statistically significant differences between the CHD group and controls.

**Table 2:** Plasma PFAS distribution in congenital heart disease cases and controls.

PFAS^a^	Congenital heart disease group (*N* = 282)					Control group (*N* = 282)					*p* value
	Mean	SD	P25	Median	P75	Mean	SD	P25	Median	P75	
6:2 ClPFESA	2.789	4.151	0.692	1.476	3.212	1.091	1.338	0.379	0.658	1.315	<0.001
8:2 ClPFESA	0.053	0.117	0.008	0.019	0.043	0.015	0.039	0.000	0.004	0.010	<0.001
FOSA	0.022	0.028	0.009	0.015	0.027	0.020	0.022	0.007	0.014	0.023	0.050
PFBA	0.223	0.884	0.062	0.119	0.221	0.024	0.041	0.002	0.004	0.033	<0.001
PFBS	0.214	0.135	0.123	0.186	0.275	0.152	0.110	0.081	0.116	0.186	<0.001
PFDA	1.088	1.490	0.305	0.616	1.312	0.861	1.083	0.312	0.561	0.944	0.197
PFHpS	0.122	0.104	0.048	0.085	0.164	0.094	0.084	0.042	0.074	0.121	0.252
PFHxA	0.018	0.010	0.010	0.016	0.023	0.013	0.014	0.006	0.009	0.015	<0.001
n-PFHxS	2.629	4.904	0.701	1.097	2.087	1.686	2.521	0.501	1.072	1.715	0.810
PFNA	1.749	1.522	0.677	1.258	2.367	1.400	1.163	0.617	1.138	1.787	0.220
PFOA	12.849	9.728	6.453	10.360	16.591	9.146	5.342	5.791	8.544	11.908	0.003
n-PFOS	5.181	6.700	1.370	2.987	6.296	3.832	4.161	1.537	2.701	4.553	0.095
PFPeA	0.007	0.008	0.003	0.005	0.009	0.005	0.005	0.001	0.002	0.006	<0.001
PFTeDA	0.055	0.052	0.021	0.043	0.070	0.032	0.050	0.006	0.014	0.035	<0.001
PFUdA	0.609	0.704	0.208	0.380	0.758	0.524	0.559	0.170	0.386	0.670	0.761
6 m-PFOS	0.452	0.439	0.169	0.305	0.582	0.394	0.404	0.169	0.272	0.496	0.187
3,4,5 m-PFOS	0.778	0.671	0.352	0.523	0.956	0.667	0.581	0.352	0.523	0.777	0.958
1 m-PFOS	0.148	0.120	0.063	0.107	0.195	0.114	0.072	0.066	0.098	0.142	0.030
Br-PFHxS	0.170	0.417	0.024	0.054	0.138	0.056	0.069	0.025	0.037	0.058	<0.001

PFAS, Per- and polyfluoroalkyl substances; P, Percentile; SD, Standard Deviation.

a For full chemical names, refer to Table 1.

### 3.2 Logistic regression analysis

**Figure 1:**
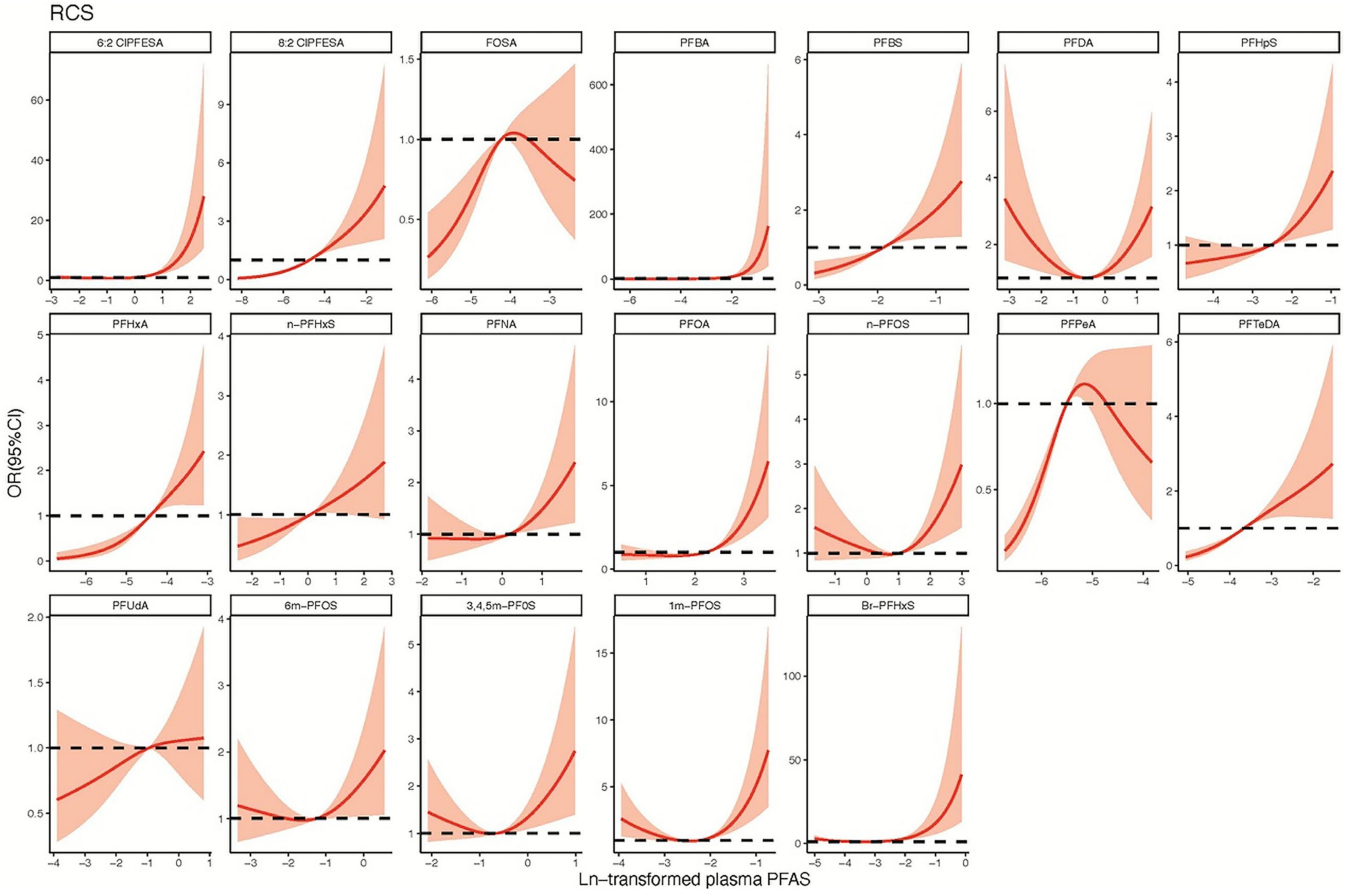
RCS curve analysis of the association between various PFAS compounds (ln-transformed) and the risk of congenital heart disease. The graph presents restricted cubic spline (RCS) curves illustrating the association between a range of perfluoroalkyl and polyfluoroalkyl substances (PFAS) and the risk of congenital heart disease. Each subplot represents a different PFAS compound, with the *x*-axis showing the log-transformed concentration of PFAS in plasma, and the y-axis displaying the odds ratio (OR) for congenital heart disease risk along with its 95% confidence interval (CI). The red shaded area indicates the range of the confidence interval. These curves reveal the nonlinear relationships between plasma PFAS concentrations and congenital heart disease risk.

Logistic regression identified significant associations between CHD risk and specific PFAS. For instance, the odds ratio (OR) for 6:2 Cl-PFESA is 1.65 (95% CI: 1.40, 1.94), indicating a 65% increase in CHD risk for each unit increase in the ln-transformed concentration of this compound. Similarly, 8:2 Cl-PFESA shows an OR of 1.92 (95% CI: 1.69, 2.19), representing a 92% increase in risk. PFBA, PFBS, PFHxA, and PFTeDA exhibit substantial ORs of 2.68, 2.38, 2.62, and 2.18, respectively, indicating strong associations with CHD risk (*p* < 0.001). Conversely, PFDA, PFUdA, 6 m-PFOS, 345 m-PFOS, and 1 m-PFOS demonstrate ORs close to 1 and non-significant *p*-values, suggesting no strong evidence of an association with CHD risk for these compounds (Table 3). Furthermore, the RCS curve depicted in Figure 1 illustrates a nonlinear relationship between PFOS and the risk of heart disease. Notably, these curves predominantly display an approximately linear shape or an inverted J-shaped pattern, signifying a notable escalation in the risk of heart disease with increasing concentrations of the compound.

**Table 3:** Associations of ln-transformed PFAS with the risk of congenital heart disease in logistic regression model (*n* = 564).

PFAS^a^	OR (mean and 95%CI) ^b^	*p*-value
6:2 ClPFESA	1.65 (1.40, 1.94)	<0.001
8:2 ClPFESA	1.92 (1.69, 2.19)	<0.001
FOSA	1.31 (1.07, 1.60)	0.010
PFBA	2.68 (2.28, 3.15)	<0.001
PFBS	2.38 (1.77, 3.22)	<0.001
PFDA	1.01 (0.86, 1.19)	0.870
PFHpS	1.38 (1.14, 1.67)	<0.001
PFHxA	2.62 (1.99, 3.45)	<0.001
n-PFHxS	1.30 (1.11, 1.53)	<0.001
PFNA	1.27 (1.04, 1.55)	<0.001
PFOA	1.64 (1.27, 2.13)	<0.001
n-PFOS	1.12 (0.95, 1.31)	0.180
PFPeA	2.11 (1.70, 2.61)	<0.001
PFTeDA	2.18 (1.81, 2.63)	<0.001
PFUdA	1.13 (0.96, 1.33)	0.150
6 m-PFOS	1.13 (0.94, 1.35)	0.210
3,4,5 m-PFOS	1.19 (0.94, 1.51)	0.150
1 m-PFOS	1.22 (0.97, 1.53)	0.100
Br-PFHxS	1.37 (1.17, 1.60)	<0.001

PFAS, Per- and polyfluoroalkyl substances; OR, Odds Ratio; CI, Confidence Interval.

a For full chemical names, refer to Table 1.

b Logistic regression models were utilized to assess the OR and 95% CI of congenital heart disease, estimated by a unit higher difference in ln-transformed PFAS as continuous variables.

### 3.3 Bayesian kernel machine regression analysis

**Figure 2:**
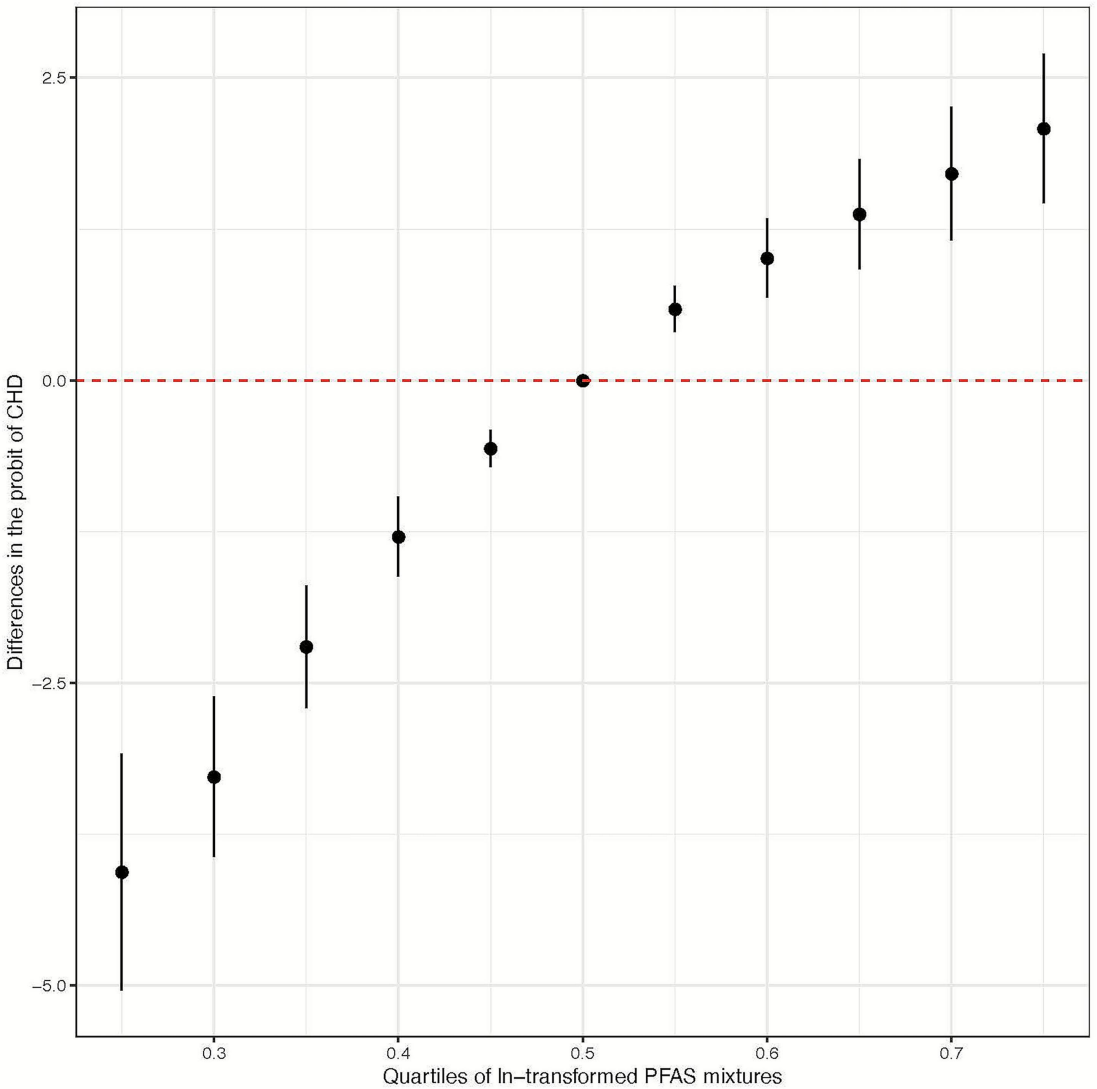
Comprehensive joint associations of PFAS mixture (ln-transformed) with the risk of congenital heart disease, evaluated via Bayesian Kernel Machine Regression (BKMR) (*n* = 564). All estimates adjusted for age (continuous) and sex (categorical). This figure illustrates the estimated difference in the probit of congenital heart disease across specific exposure percentiles (*x*-axis) compared to when all exposures are at the 50th percentile.

**Figure 3:**
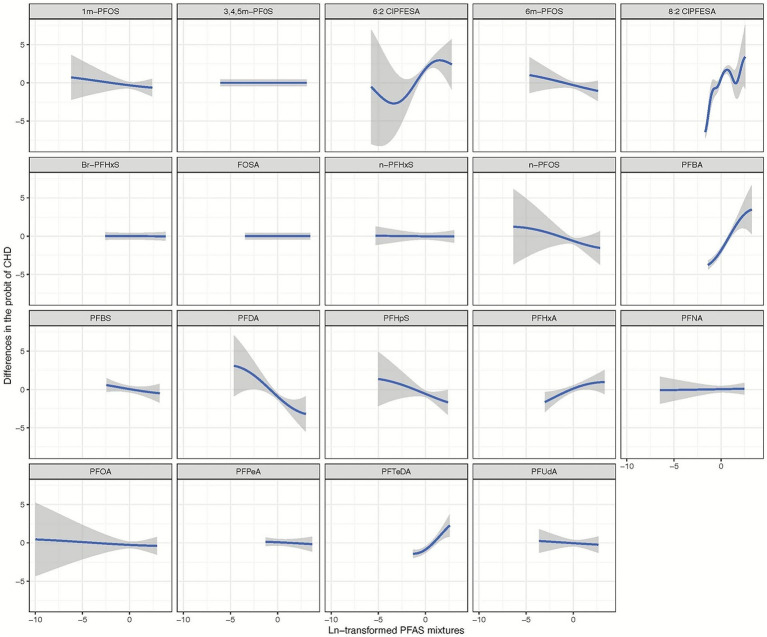
Univariate exposure-response relationship of individual ln-transformed PFAS with the risk of congenital heart disease, estimated by Bayesian Kernel Machine Regression (BKMR) for each PFAS, with the other pollutants fixed at the median (*n* = 943). All estimates were adjusted for age (continuous), sex (categorical). The boundaries of the gray areas represent the 95% CIs of the exposure-response relationship.

Figure 2 illustrates the comprehensive joint associations of PFAS mixture exposure with the risk of CHD as evaluated by BKMR. This trend suggests that higher levels of PFAS mixture exposure are significantly associated with an increased risk of CHD. Additionally, The BKMR model identified significant contributors to the overall association, including 6:2 Cl-PFESA, 8:2 Cl-PFESA, PFBA, PFHxA, and PFTeDA, all with PIPs of 1.00. A PIP of 1.00 indicates that these variables were consistently included in all iterations of the model, suggesting their strong and reliable association with CHD risk (refer to Figure 3; Supplementary Table S2).

### 3.4 Weighted quantile sum regression

**Figure 4:**
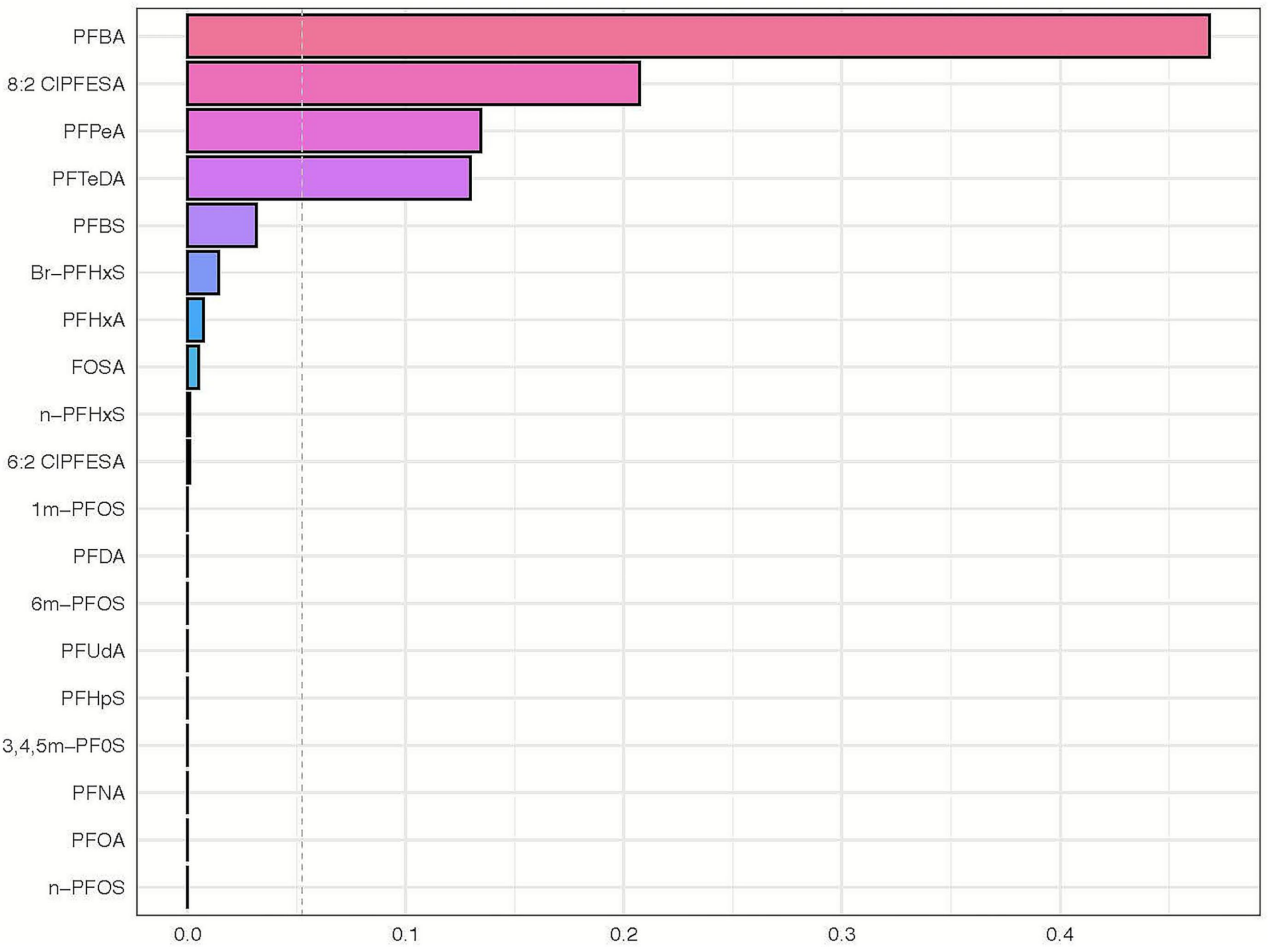
Weighted quantile sum regression analysis of PFAS mixture exposure and its association with congenital heart disease. This horizontal bar chart represents the weighted quantile sum (WQS) regression analysis outcomes of various perfluoroalkyl and polyfluoroalkyl substances (PFAS) in relation to the risk of congenital heart disease. Each bar corresponds to a specific PFAS compound, with the length of the bar indicating the weight in the WQS index. The chart reveals the relative contribution of each PFAS compound to the overall risk of congenital heart disease as determined by the gWQS regression technique. Compounds with longer bars have a higher weighted impact in the model, suggesting a more significant association with the risk of congenital heart disease.

The results of the WQS analysis are presented in Figure 4. Overall, PFAS mixture exposure was associated with an increased risk of CHD, with an odds ratio of 3.49 (Standard Error: 0.25, *q* < 0.001) for each quantile of PFAS concentration. In our WQS regression analysis, the OR of 3.49 represents the increase in the odds of CHD associated with a one-quartile increase in the weighted sum of PFAS exposures. Specifically, this means that for each quartile increase in the overall PFAS mixture exposure (as quantified by the WQS index), the odds of CHD increase by 249% (OR-1 = 2.49, or 249%), after adjusting for covariates. The weights assigned to each PFAS compound in the WQS model represent their relative importance in the mixture’s association with CHD risk. These weights were consistently distributed across quantiles, with PFBA consistently receiving the highest weight (mean weight: 0.436), followed by 8:2 Cl-PFESA (0.256), PFTeDA (0.123), and PFHxA (0.101). This distribution remained stable across bootstrap iterations, suggesting a robust pattern of relative importance among the PFAS compounds. (refer to Figure 4; Supplementary Table S2).

## 4 Discussion

This investigation underscores the association of various PFAS compounds, including traditional and emerging ones, with CHD incidence in children. Specifically, 6:2 Cl-PFESA, 8:2 Cl-PFESA, PFBA, PFHxA, and PFTeDA emerged as significant factors in this association. These findings highlight the importance of considering the full range of PFAS compounds to fully understand the health effects of environmental exposure. The strength of our study lies in its constructed hospital-based case–control design. While multiple PFAS compounds were analyzed using advanced statistical methods, our analyses enhance understanding of their potential health effects. These insights represent a crucial addition to the growing body of evidence linking PFAS exposure to harmful cardiovascular effects, providing valuable guidance for developing public health policies and initiatives aimed at reducing exposure and preventing CHD in children.

Our study expands upon existing research by investigating the association between PFAS exposure and cardiovascular disease, specifically focusing on CHD in children. While previous studies concentrated on traditional PFAS, our research broadens the scope to include emerging compounds, enhancing understanding of their impact on cardiovascular health, particularly childhood CHD. We conducted a hospital-based case–control study involving 282 children with CHD and an equal number of controls, meticulously analyzing plasma samples for 19 PFAS compounds, encompassing long-chain and short-chain PFAS, as well as emerging alternatives and branched-chain isomers. Utilizing two multi-pollutant models (WQS and BKMR), we assessed the association between individual PFAS and PFAS mixtures with CHD. Both models consistently indicated a positive correlation, suggesting that exposure to a combination of PFAS compounds may increase CHD risk in children. After adjusting for other PFAS congeners, five specific compounds, 6:2 Cl-PFESA, 8:2 Cl-PFESA, PFBA, PFHxA, and PFTeDA, emerged as major contributors to the association between PFAS mixtures and CHD, implying significant roles for these compounds in CHD development.

Our findings are consistent with previous research, including two Chinese studies suggesting that gestational exposure to most PFAS is associated with a higher risk of CHD (20, 23). However, a comparison of the specific PFAS species involved reveals important differences. The studies by Li et al. (20) and Ou et al. (23) primarily identified long-chain perfluoroalkyl carboxylic acids (PFCAs), whereas our study, in addition to some long-chain compounds (PFTeDA), identified significant roles for emerging alternatives (e.g., 6:2 and 8:2 Cl-PFESA) and short-chain PFAS (e.g., PFBA, PFHxA). The widespread presence of PFAS in the environment, detectable in air, water, soil, and human blood, underscores their bioaccumulative potential and raises significant public health concerns regarding their long-term impact. This study reinforces the association between PFAS exposure and cardiovascular pathology and underscores the urgent need for a comprehensive investigation into the impact of a broader range of PFAS compounds, especially on vulnerable groups such as children.

The mechanisms through which PFAS contribute to CHD development remain unclear but may involve several potential pathways. One notable mechanism is endocrine disruption, where PFAS interfere with critical hormone regulation, such as thyroid hormones essential for heart development (17, 30, 31). Disruptions in thyroid hormone levels during fetal development stages could lead to structural heart abnormalities, increasing CHD risk (32, 33). Another proposed pathway involves oxidative stress and inflammation (9, 34, 35). PFAS trigger oxidative stress by disrupting the balance between reactive oxygen species (ROS) production and the body’s antioxidative defenses, leading to cellular and tissue damage, including in the developing heart (9, 36). Additionally, PFAS can activate inflammatory pathways, exacerbating tissue damage and hindering normal heart formation (19, 37). PFAS are also implicated in altering lipid metabolism, leading to dyslipidemia—a recognized risk factor for cardiovascular diseases, including CHD (38, 39). Furthermore, disturbances in calcium homeostasis, crucial for cardiac muscle function, may result from PFAS exposure, potentially leading to abnormal heart rhythms and compromised cardiac function, further increasing CHD risk (12, 19). PFAS exposure has also been associated with changes in the expression of genes crucial for cardiac development, potentially directly contributing to structural heart abnormalities and elevated CHD risk (23, 40–42) 6:2 Cl-PFESA.

These mechanisms are likely interrelated and may collectively influence CHD development. Additionally, the impact of specific PFAS compounds and the timing of exposure during developmental stages may dictate the extent and nature of these effects. In summary, PFAS contribute to childhood CHD development through multiple intertwined pathways. Legacy long-chain PFASs contributed mostly to endocrine disruption, and short-chain PFASs were more strongly linked to its potential to induce oxidative stress and disrupt mitochondrial function. While the chlorinated PFASs (6:2 and 8:2 Cl-PFESA) are likely related to endocrine disruption, the disruption of calcium homeostasis and gene expression changes. Further research is essential to elucidate these complex interactions and their implications for child health.

Our study findings have significant implications for both public health policy and clinical practice. The established association between PFAS exposure and increased CHD risk in children calls for a reassessment of public health strategies and regulatory measures to limit environmental exposure to these substances, especially among vulnerable groups like children. Additionally, our findings emphasize the need for comprehensive risk assessments and regulatory policies covering the full spectrum of PFAS chemicals. By identifying specific contributors, we offer actionable insights for crafting targeted public health interventions aimed at reducing environmental exposure to these chemicals and mitigating associated pediatric cardiovascular risk. In the clinical domain, our findings are equally important. Healthcare practitioners should integrate our insights into their risk assessment protocols and patient care strategies, recognizing the potential impact of PFAS exposure on pediatric cardiac development. Enhanced understanding of how environmental factors like PFAS contribute to CHD can inform clinical decision-making and patient management, leading to improved outcomes for affected individuals. It is imperative for healthcare providers to consider environmental determinants of CHD to facilitate early detection and intervention, ensuring optimal care provision for children with or at risk of this condition. Furthermore, our study lays the foundation for future research to investigate the mechanisms underlying PFAS exposure’s effects on cardiovascular health and its long-term implications. This knowledge is crucial for refining risk assessment methodologies and developing precise preventive measures, thereby enhancing public health strategies and individual patient care on a broader scale.

While our study provides valuable insights, it is important to acknowledge its limitations. Firstly, the cross-sectional design restricts our ability to establish a causal relationship between PFAS exposure and CHD onset. Prospective longitudinal studies are necessary to validate our findings and determine causality. Secondly, our research focused on a specific population within China, potentially limiting the generalizability of our results to other geographical areas and demographic groups. Additionally, our study encountered limitations regarding data availability, particularly in assessing other influential factors such as genetic predispositions or concurrent environmental exposures that could influence the PFAS-CHD relationship. Furthermore, data on potential confounders like maternal exposures during pregnancy, specific dietary patterns, and detailed family socioeconomic status were not available. These unexplored confounders may impact the observed associations, underscoring the importance of comprehensive data collection in future investigations. Finally, the intricate mechanisms by which PFAS exposure affects childhood CHD development remain incompletely understood. Future research should strive to elucidate these mechanisms, providing a clearer understanding of the biological basis of PFAS-related cardiac anomalies.

## 5 Conclusion

In conclusion, our study underscores the observed association between PFAS exposure and increased CHD risk in children, with specific focus on the impact of 6:2 Cl-PFESA, 8:2 Cl-PFESA, PFBA, PFHxA, and PFTeDA. Utilizing advanced statistical methods, we conducted a comprehensive analysis of various PFAS compounds, identifying key contributors and providing suggestive evidence for this critical association. These findings underscore the urgency of implementing stringent regulations on PFAS to safeguard public health. Moving forward, research efforts should prioritize unraveling the mechanisms linking PFAS exposure to CHD, enabling the development of targeted interventions aimed at reducing exposure and preventing adverse health outcomes.

## Data Availability

The raw data supporting the conclusions of this article will be made available by the authors, without undue reservation.
