# Supplementary material for: Environmental exposure to per- and polyfluoroalkyl substances and childhood congenital heart disease: a mixed analysis
Source: Front Public Health. 2025 Nov 27;13:1657168. doi: 10.3389/fpubh.2025.1657168 (PMC12695596; doi:10.3389/fpubh.2025.1657168)
Supplement: Supplementary file 1 [file Table_1.docx]

**Table S1. Characteristics of participants in this study.**

| **Characteristics** | **Total (N = 564)** | **Congenital heart disease group (N = 282)** | **Control group (N = 282)** |
| --- | --- | --- | --- |
| Age [y,mean (SD)] | 7.3 (2.3) | 7.1 (2.5) | 7.5 (2.1) |
| Sex [n (%)] |  |  |  |
| Boy | 350 (62.1) | 178 (63.1) | 172 (61.0) |
| Girl | 214 (37.9) | 104 (36.9) | 110 (39.0) |

**Table S2. Estimated posterior inclusion probability (BKMR analyse) and weight in the WQS index (WQS analyse) for PFAS in relation to the risk of congenital heart disease (n = 564).**

| **PFAS** | **PIP** | **Mean weight** |
| --- | --- | --- |
| 6:2 ClPFESA | 1.000 | 0.007 |
| 8:2 ClPFESA | 1.000 | 0.256 |
| FOSA | 0.000 | 0.002 |
| PFBA | 1.000 | 0.436 |
| PFBS | 0.534 | 0.050 |
| PFDA | 1.000 | 0.000 |
| PFHpS | 0.976 | 0.000 |
| PFHxA | 1.000 | 0.021 |
| n-PFHxS | 0.028 | 0.000 |
| PFNA | 0.028 | 0.000 |
| PFOA | 0.310 | 0.000 |
| n-PFOS | 0.480 | 0.000 |
| PFPeA | 0.376 | 0.101 |
| PFTeDA | 1.000 | 0.123 |
| PFUdA | 0.138 | 0.000 |
| 6m-PFOS | 0.498 | 0.000 |
| 3,4,5m-PFOS | 0.000 | 0.000 |
| 1m-PFOS | 0.346 | 0.000 |
| Br-PFHxS | 0.006 | 0.003 |

Notes: PIP: posterior inclusion probability; PFAS with PIP > 0.5 were empirically regarded as important to outcome specific association. Compounds with longer bars have a higher weighted impact in the model, suggesting a more significant association with the risk of congenital heart disease.
