# Supplementary material for: Environmental exposure to per- and polyfluoroalkyl substances and childhood congenital heart disease: a mixed analysis
Source: Front Public Health. 2025 Nov 27;13:1657168. doi: 10.3389/fpubh.2025.1657168 (PMC12695596; doi:10.3389/fpubh.2025.1657168)
Supplement: Supplementary file 2 [file Supplementary_file_1.docx]

**Supplementary figures**


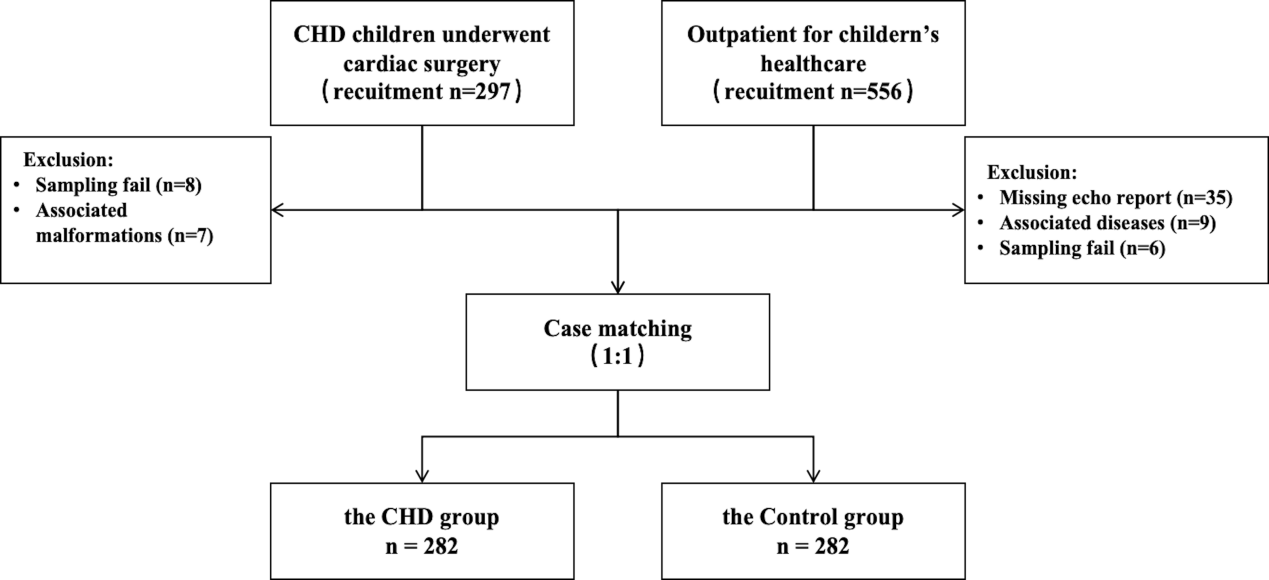


**Figure S1. Flowchart of the study participants recruitment**


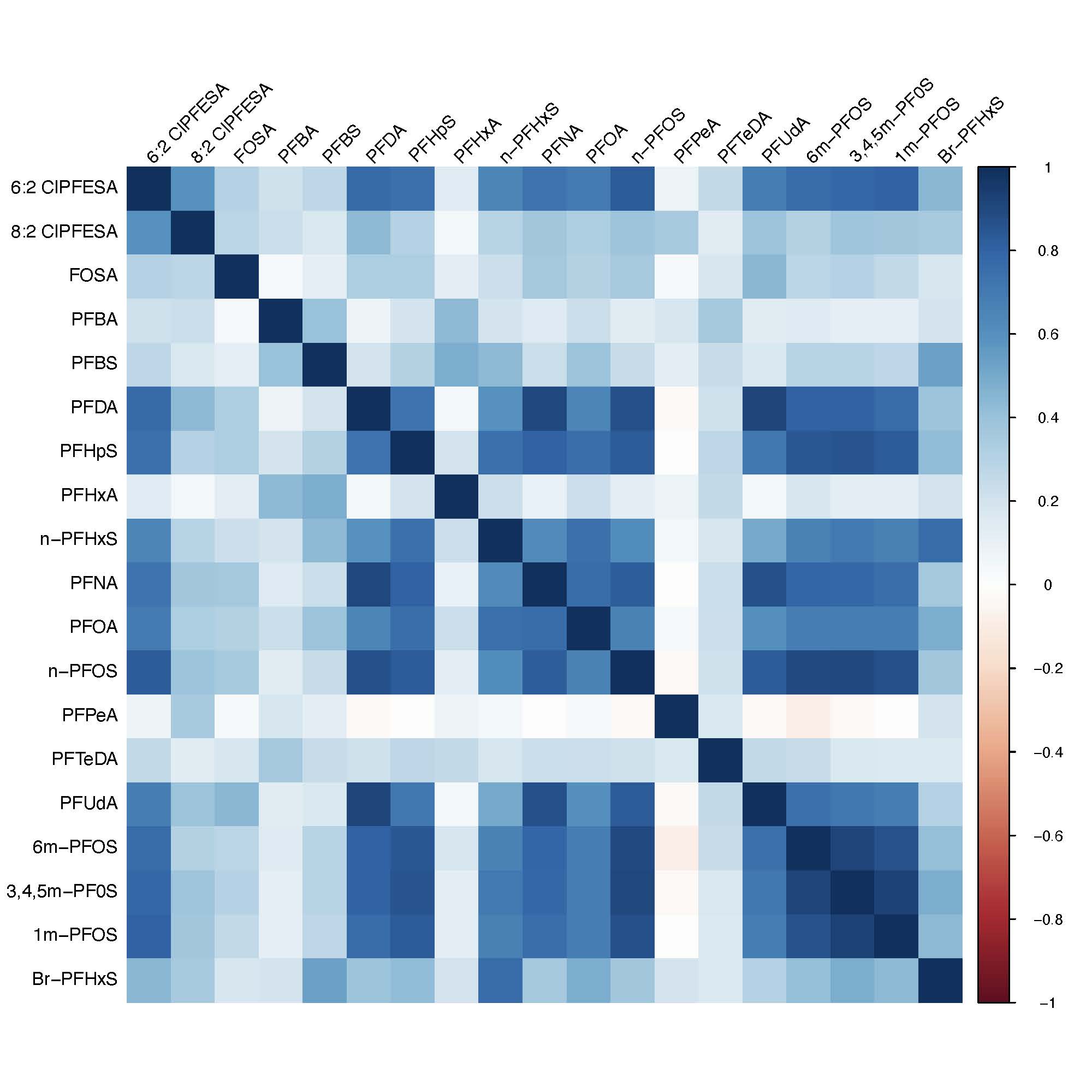


**Figure S2. Clustered heatmap of the spearman correlation matrix of PFAS (ln-transformed)** **in all cases (n = 564).**
